# Transcription factor BRCA1 is associated with BMPR1B transcriptional activity in sheep ovarian granulosa cells

**DOI:** 10.3389/fvets.2025.1722859

**Published:** 2026-01-14

**Authors:** Anwar Abdurahman, Alimujiang Abulizi, Abudukaibier Abudukeremu, Fei Zhang, Yuling Ga, Rahmantay Obulkasim

**Affiliations:** 1 Xinjiang Key Laboratory of Mental Development and Learning Science, School of Psychology, Xinjiang Normal University, Urumqi, Xinjiang, China; 2 College of Animal Science and Technology, Nanjing Agricultural University, Nanjing, China; 3 Shufu County Agriculture, and Rural Affairs Bureau, Kashi, China; 4 Aketao xian zhong deng zhi ye ji shu xue xiao, Kezilisu, China; 5 Animal Diseases Control and Prevention Centre of Xinjiang Uygur Autonomous Region, Urumqi, China; 6 Ili Kazak Autonomous Prefecture Institute of Animal Science, Ili, China

**Keywords:** BMPR1B, BRCA1, ovarian GCs, sheep, transcription factor

## Abstract

BMPR1B is a well-characterized major gene associated with prolificacy in sheep. The FecB mutation in this gene promotes accelerated follicular development and increases ovulation rate, thereby enhancing litter size. Although BMPR1B plays a crucial role in reproduction, the regulatory mechanisms of its expression in sheep tissues are not yet fully understood. This study identified the promoter region of the BMPR1B gene and provided evidence that its transcriptional activity is associated with regulation by the transcription factor BRCA1. Luciferase reporter assays showed that the P1 fragment containing the BMPR1B promoter region had gene expression-driving activity; importantly, two BRCA1 binding sites (BBS) were identified within this promoter region. Subsequent experimental results suggest that BRCA1’s effect on BMPR1B transcriptional activity may be achieved through BBS2 rather than BBS1. Further studies in sheep ovarian granulosa cells (GCs) have shown that BRCA1 expression is correlated with BMPR1B expression and is linked to reduced granulosa cell apoptosis. Collectively, these findings provide a basis for the BRCA1-BMPR1B regulatory axis and offer new insights into the molecular framework influencing ovarian granulosa cell apoptosis in sheep.

## 1 Introduction

Bone morphogenetic proteins (BMPs) are members of the transforming growth factor-*β* (TGF-β) superfamily and trigger signal transduction by heterodimerizing a type I receptor (BMPR1B/ALK6) with a type II receptor (1). After a ligand engages the receptor complex, the constitutively active type II serine/threonine kinase transfers a phosphate group to the adjacent type I receptor. Consequently, the activated type I receptor catalyzes the phosphorylation of the receptor-regulated R-SMAD proteins. Phosphorylation-activated R-SMAD proteins bind to the common-mediator SMAD4, forming a heterotrimeric assembly that subsequently migrates into the nucleus to control the transcription of genes such as Runx2 and Osterix (2, 3). The BMP/SMAD pathway constitutes a pivotal signal-transduction cascade that governs cell differentiation (4), proliferation (5), and apoptosis (6). In ovine reproduction, this pathway exerts essential functions in ovulation and oocyte maturation (7).

BMPR1B serves as a key type-I receptor in the bone morphogenetic protein/SMAD signaling cascade, and alterations in its expression level can modulate pathway activity (8). By interacting with BMP ligands, BMPR1B governs the proliferation, differentiation, and programmed cell death that directly determine ovulation rate. Consequently, reproductive efficiency in sheep is largely determined by this mechanism (9). Segregation analyses have revealed that the B allele of BMPR1B increases litter size; homozygous BB females first appeared in the F1 generation and produced significantly more lambs than ++ ewes (10–12). These findings establish BMPR1B as a major gene for ovulation rate and suggest that distinct molecular mechanisms downstream of the receptor fine-tune prolificacy, thereby providing selectable molecular markers for sheep breeding. GCs are stratified epithelial cells that surround the oocyte within the ovarian follicle and orchestrate folliculogenesis; hence, their functional integrity is indispensable for fertility (13). The BMPR1B gene constitutes a key regulatory node in granulosa cell function. Our laboratory previously demonstrated that, in sheep ovarian GCs, the transcription factor SMAD4 exerts feedback regulation that enhances BMPR1B transcription (6). Another study from our group identified miR-1306 as a direct regulator of BMPR1B in sheep GCs, leading to the induction of apoptosis (7).

Breast cancer-associated gene 1 (BRCA1) was the first breast cancer susceptibility gene identified on human chromosome 17q12-21. Mutations in the BRCA1 gene constitute a major hereditary risk factor for both ovarian and breast cancers (14–16). The development and advancement of ovarian carcinoma are tightly associated with the control of the cell-cycle and the effective activation of apoptosis pathways (17). As a pivotal tumor-suppressor gene, BRCA1 is a key regulator of cell growth and programmed cell death in mammalian cells (18). Recent studies indicate that non-coding RNAs affect the expression of BRCA1 together with its partner BARD1, thereby regulating steroid hormone production in ovarian GCs and revealing an additional mechanism by which BRCA1 contributes to ovarian tumorigenesis (19–21). Oxidative stress regulated by the miR-1307/BRCA1 pathway triggers programmed cell death in porcine ovarian GC, underscoring the importance of BRCA1 in maintaining cell-cycle homeostasis and inhibiting excessive apoptosis (22), a role crucial for preserving the equilibrium of cell-cycle progression and suppressing programmed cell death.

During the regulation of gene expression, transcription factors recruit RNA polymerase II to form a transcriptional assembly, which then engages the specific promoter regions of their target genes, thereby either activating or repressing the transcription of downstream genes. Consequently, transcription factors exert a pivotal regulatory influence on transcriptional output (23). BRCA1 is closely associated with genomic-maintenance functions such as DNA repair and the enforcement of cell-cycle checkpoints. BRCA1 works together with RAD51, BRCA2, and PALB2, as well as additional repair factors, to promote the precise restoration of double-strand DNA breaks (24). Via its BRCT domain, BRCA1 associates with the Pol II holocomplex and activates the expression of genes such as GADD45 and p21. By promoting p53-mediated G1 arrest, BRCA1 enhances cell-cycle inhibition, while p53, in turn, negatively regulates BRCA1 expression, establishing a dynamic feedback loop (25).

Our previous investigations revealed that miR-1306 triggers programmed cell death in ovine GCs through a reverse-complementary interaction with the 3′-UTR of the BMPR1B gene (7). Despite its significance, the regulatory mechanisms governing transcription of the ovine BMPR1B gene remain poorly understood. In the present work, we show that the transcription factor BRCA1 suppresses apoptosis of sheep ovarian GCs by binding to a specific motif in the 5′-regulatory region of the BMPR1B gene, thereby enhancing its transcriptional activity.

## 2 Materials and methods

### 2.1 Sample collection

Ear tissue samples from Hu sheep were kindly provided by the Xilaiyuan Breeding Farm located in Taizhou, China, for genomic DNA isolation. Additionally, the Hualing slaughterhouse (Urumqi, Xinjiang) served as the source for the fresh ovarian samples. These ovarian samples were then used to isolate the GC. All procedures involving animals received approval from the Animal Care and Use Committee of Nanjing Agricultural University, ensuring full compliance with China’s Experimental Animal Administration Regulations (State Science and Technology Commission Decree No. 2, enacted on November 14, 1988; approval code SYXK 2017–0027).

### 2.2 Cell culture

KGN and HEK293T cell lines, utilized in the dual-luciferase assays, were acquired from the Shanghai Cell Bank of the Chinese Academy of Sciences. The handling and transfection of these cells strictly followed a protocol we have described previously (26). For the preparation of primary ovine GCs, ovaries were transported from the slaughterhouse to the laboratory within a two-hour window and maintained at 37 °C in sterile saline solution. The isolation and culture of GCs from these ovaries were then performed based on an established methodology cited elsewhere (27, 28).

### 2.3 Cell transfection

For transfection experiments, HEK293T and KGN cells were seeded into 12-well plates and grown for 24 h to reach 70–80% confluence. The cells were then transfected with the respective plasmids (pcDNA3.1-BRCA1 overexpression vector, pGL3-Basic promoter reporter constructs) using Lipofectamine 3,000 (Invitrogen, USA) according to the manufacturer’s instructions. A total amount of 1.0 μg of plasmid DNA was used for each transfection in a 12-well plate, with a ratio of 1:1 when co-transfecting two different plasmids. The culture medium was replaced with fresh complete medium 6 h post-transfection, and cells were collected for subsequent analyses after 24–48 h.

### 2.4 RNA/DNA extraction and quantitative real-time PCR

Genomic DNA from sheep tissues was purified via the standard phenol-chloroform extraction method. Concurrently, total RNA was isolated from GC using TRIzol® reagent (Invitrogen, USA), adhering to the supplier’s guidelines. The synthesis of complementary DNA (cDNA) from the extracted RNA was carried out with PrimeScript™ RT Master Mix (TaKaRa, China). To assess gene expression, quantitative real-time PCR (qRT-PCR) was run in triplicate using SYBR® Premix Ex Taq (TaKaRa), with GAPDH functioning as the endogenous control for normalization. The 2^-ΔΔCt^ algorithm was applied to calculate relative expression levels.

### 2.5 Plasmid construction

To identify the promoter region of the Hu sheep BMPRIB gene (NC_019463.2), we utilized a deletion expression approach to amplify its 5’flanking sequence. As detailed in Supplementary Table 1, the primers’ design included the addition of SacI and XhoI endonuclease sites to the 5′ and 3′ ends, in that order. An overexpression plasmid, pcDNA3.1-BRCA1, was generated in our laboratory. The PCR products underwent purification using the Axy prepTM PCR cleanup kit (Axygen, Union City, CA, USA). Subsequently, these products were sub-cloned into the pGL3 luciferase reporter vector, which was then introduced into *E. coli* to create the luciferase reporter plasmids. Plasmids were extracted using an Endo-free plasmid mini kit II50 (Omega, Norcross, GA, USA) and subsequently validated through sequencing. Furthermore, BBS mutation-typed plasmids were created utilizing the TaKaRa MutanBEST Kit (TaKaRa). The pcDNA3.1-BRCA1 plasmid was constructed previously by our team.

### 2.6 Dual luciferase assay

For the reporter assays, HEK293T and KGN cells were seeded at a density of 2.0 × 10^5^ cells per well and grown for 24 h to reach appropriate confluence. Cells were then co-transfected with 1.2 μg of total plasmid DNA per well. This DNA comprised pGL3-Basic reporter constructs (or deletion/mutation variants), the pcDNA3.1-BRCA1 overexpression plasmid (or the empty pcDNA3.1 vector), and the pRL-TK vector (Promega), combined at a mass ratio of 10:1:1. The transfection was performed using Lipofectamine 3,000 transfection reagent (Invitrogen) in accordance with the manufacturer’s instructions. The pRL-TK vector, responsible for expressing Renilla luciferase, functioned as an internal normalization control.

At 24 h post-transfection, the culture medium was aspirated, and the cells were washed thrice with phosphate-buffered saline (PBS). Cell lysis was then conducted using the passive lysis buffer provided in the kit, with thorough pipetting to ensure complete disruption. After a brief centrifugation to pellet debris, the supernatant was collected. Luciferase activities (both Firefly and Renilla) in the cleared lysates were measured sequentially with the Dual-Luciferase Reporter Assay System (Abcam, #E1910) on a microplate reader, following the manufacturer’s protocol.

### 2.7 Bioinformatics analysis

To identify potential promoter regions, we utilized the online Promoter 2.0 Prediction Server (accessible at https://services.healthtech.dtu.dk/services/Promoter-2.0/). Afterwards, we scanned for transcription-factor binding motifs by querying the JASPAR database (accessible at https://jaspar.elixir.no/), which supplies expertly curated position-frequency matrices for motif detection.

### 2.8 Western blot

Cellular proteins were extracted from GC using RIPA lysis buffer, and their concentrations were determined with a BCA assay kit (Thermo Fisher Scientific, USA). Subsequent immunoblotting steps were performed as reported earlier (7). Briefly, membranes were incubated with primary antibodies targeting BMPR1B (Abcam, ab155058; 1:1500) and GAPDH (Abcam, ab9482; 1:1500), followed by incubation with species-appropriate horseradish peroxidase (HRP)-conjugated secondary antibodies (Origene; 1:1500). Protein band signals were visualized and quantified densitometrically using ImageJ software.

### 2.9 Apoptosis analysis

Apoptosis of GC was assessed by flow cytometry using an Annexin V-FITC/PI apoptosis detection kit, as previously described (7).

### 2.10 Statistical analysis

The data are presented as the mean ± SEM, derived from a minimum of three distinct biological replicates. The precise number of samples (n) for each experiment can be found in the figure legends. An unpaired two-tailed Student’s t-test was used for statistical comparisons between two groups, while one-way ANOVA followed by Tukey’s *post hoc* test was employed for comparisons involving more than two groups. These parametric tests were utilized after verifying that the data satisfied the assumptions of normality (using the Shapiro–Wilk test) and equal variance (using Levene’s test). All analyses were conducted using SPSS 27.0, with a significance threshold set at ∗*p* ≤ 0.05.

## 3 Results

### 3.1 Identification of the core promoter region of Hu sheep BMPR1B

**Figure 1:**
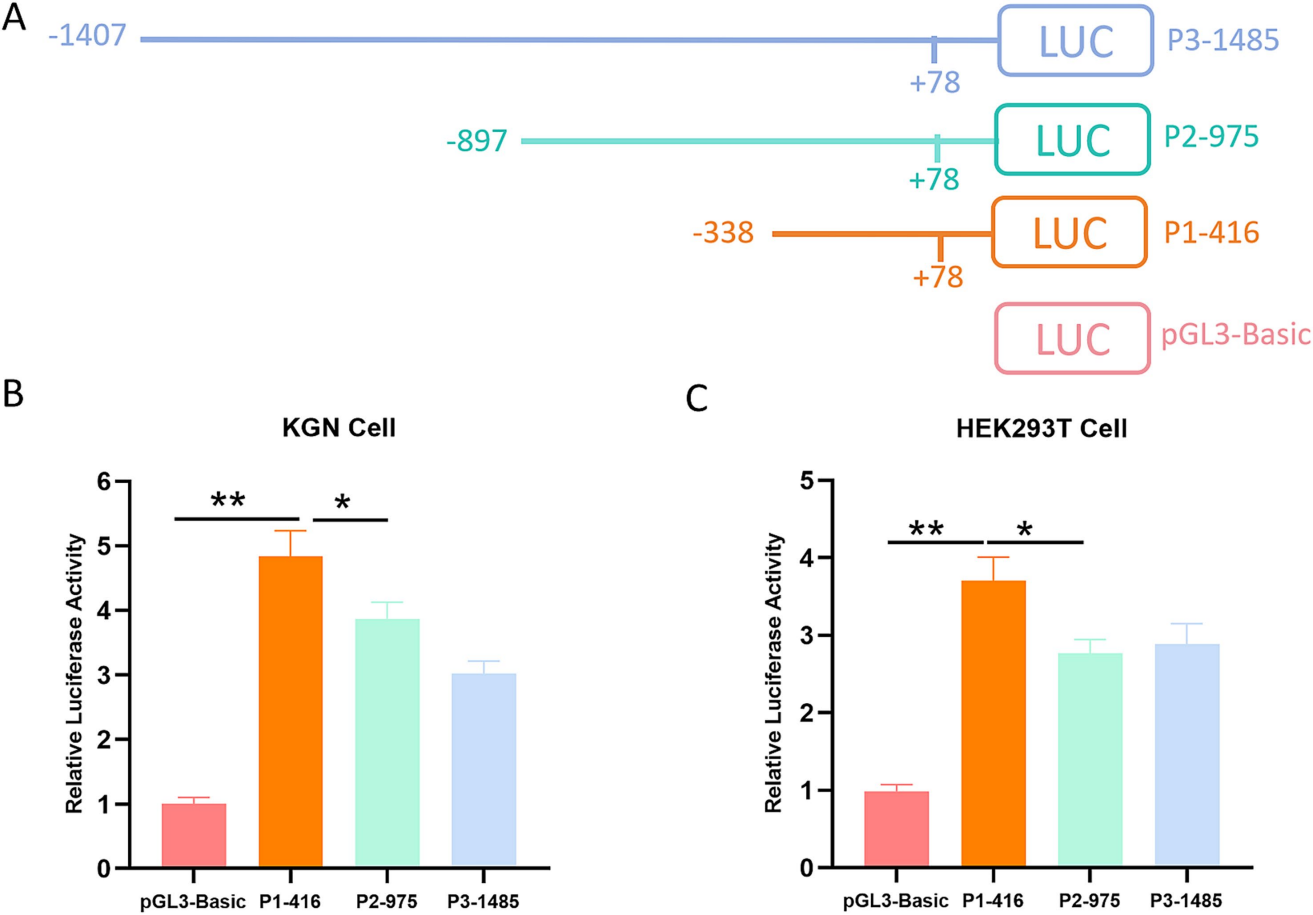
Analysis of the BMPR1B gene promoter region. **(A)** Graphical representation of the steps involved in generating the deletion expression vector. **(B,C)** Luciferase activity assay. The deletion expression vector was transfected into KGN cells **(B)** and HEK293T cells **(C)**. The luciferase activity was detected using a dual-luciferase reporter system. Each bar represents the average ± SEM (*n* ≥ 3). ^**^p ≤ 0.01, ^*^p ≤ 0.05.

**Figure 2:**
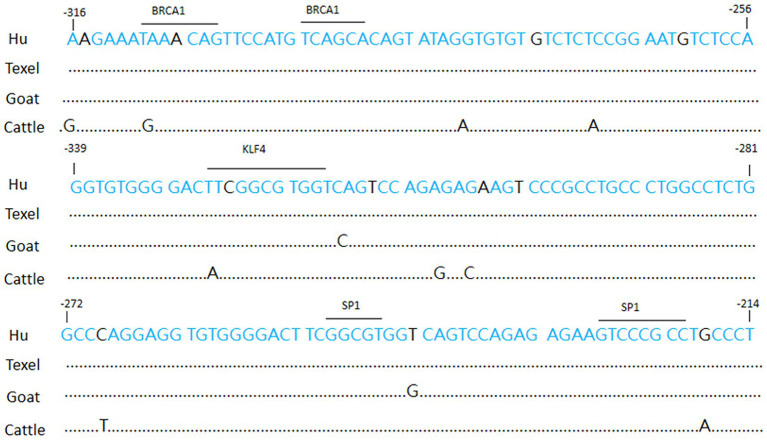
Schematic diagram of transcription factor binding on the promoter.

The promoter, which is found upstream of the gene’s coding sequence, is essential for regulating gene expression. It constitutes the initial region of transcription and contains the specific DNA sequence that is recognized and bound by RNA polymerase, which initiates the transcription process (29). To identify the promoter region, we cloned three fragments (−338/+78, −897/+78, and −1,407/+78) into the luciferase reporter vector pGL3-Basic to construct deletion expression vectors, which were named P1-416, P2-975, and P3-1485, respectively (Figure 1A). These deletion constructs and the empty pGL3-Basic vector were transiently transfected into KGN and HEK293T cells. Cells were collected after 24 h, and luciferase activity was detected by chemiluminescence. The results are presented in Figures 1B,C, 2. The trends of luciferase activity for each construct are similar in both cell lines. Notably, the P1-416 construct exhibited significantly higher activity than the empty vector and the P2 and P3 constructs, indicating that the P1 fragment contains the BMPR1B gene’s promoter region.

### 3.2 Analysis of the promoter domain of the BMPR1B gene in sheep

Gene expression is primarily regulated through the interaction of transcription factors with gene promoters. The binding of transcription factors to specific DNA sequences, known as motifs, occurs in promoter regions, thereby modulating the recruitment and activity of RNA polymerase and ultimately influencing transcriptional output. We utilized two web-based resources, JASPAR (accessible at http://jaspardev.genereg.net) and TRANSFAC (accessible at https://genexplain.com/transfac-product), to forecast the transcription factors that potentially bind to the BMPR1B promoter in Hu sheep. Our results demonstrate the presence of multiple transcription factor binding sites (e.g., for BRCA1, KLF4, and SP1) in the BMPR1B promoter region (Figure 2).

### 3.3 The promoter activity of the BMPR1B gene in Hu sheep is modulated by the transcription factor BRCA1

**Figure 3:**
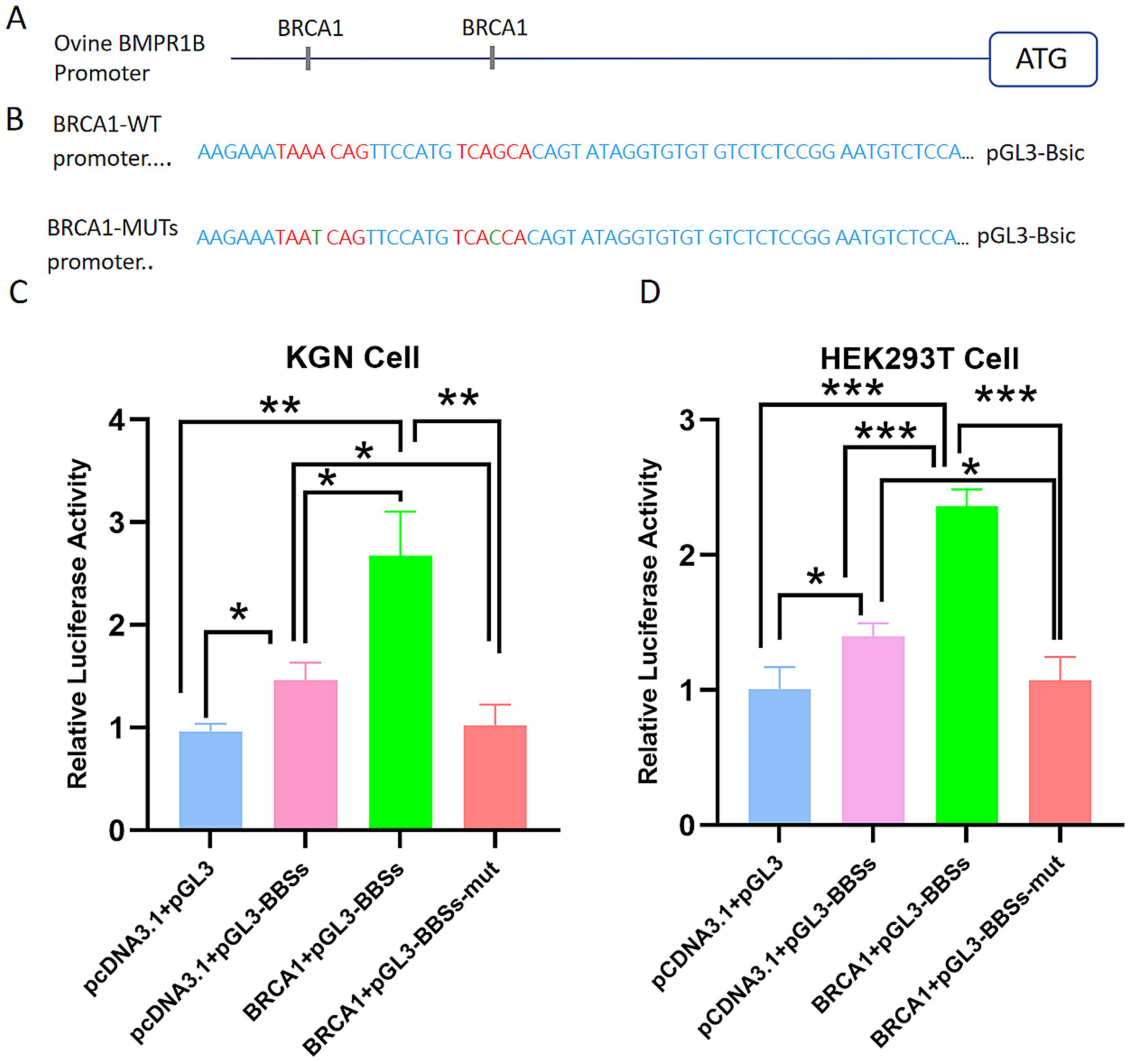
BRCA1 transcriptionally activates the BMPR1B promoter through specific binding to the BBS2 site. **(A)** Schematic diagram of BRCA1 binding sites (BBS) in the BMPR1B promoter region. **(B)** Detailed sequence diagrams showing wild-type and mutated BBS sites created by site-directed mutagenesis. **(C,D)** Relative luciferase activity in **(C)** KGN and **(D)** HEK293T cells co-transfected with either the control vector (pcDNA3.1) or BRCA1 expression vector (pcDNA3.1-BRCA1), together with the following reporter constructs: pGL3-Basic (empty vector), WT (wild-type promoter), BBS1-MUT, or BBS2-MUT. Each bar represents average ± SEM (*n* ≥ 3). *p ≤ 0.05, **p ≤ 0.01, ***p ≤ 0.001.

BRCA1 exhibits high expression levels in ovarian tissue and has been linked to key aspects of female reproduction, including ovarian reserve, oocyte quality, and fertility (30, 31). According to online software prediction, two binding sites for BRCA1 (BBSs) are located in the promoter region of the Hu sheep BMPR1B. To investigate whether BRCA1 exerts a regulatory effect on this promoter, we first constructed a reporter vector containing the Hu-sheep BMPR1B promoter (Figure 3A) and an overexpression plasmid pcDNA3.1-BRCA1. The specific sequences and locations of the two BRCA1-binding sites (BBS1 and BBS2) within the promoter region are further illustrated in Figure 3B, with their conservation and potential regulatory significance confirmed through sequence alignment and motif analysis. These constructs were co-transfected into KGN and HEK293T cells. Following a 24-h incubation, the cells were collected for the measurement of dual-luciferase activity. Overexpression of BRCA1 led to a significant increase in promoter activity in both cell lines, whereas mutation of the BBSs abolished this effect (Figures 3C,D). These results demonstrate that BRCA1 up-regulates the transcription of the BMPR1B by binding to BRCA1-binding sites located within its promoter.

### 3.4 A direct interaction occurs between the transcription factor BRCA1 and the promoter of the BMPR1B gene

**Figure 4:**
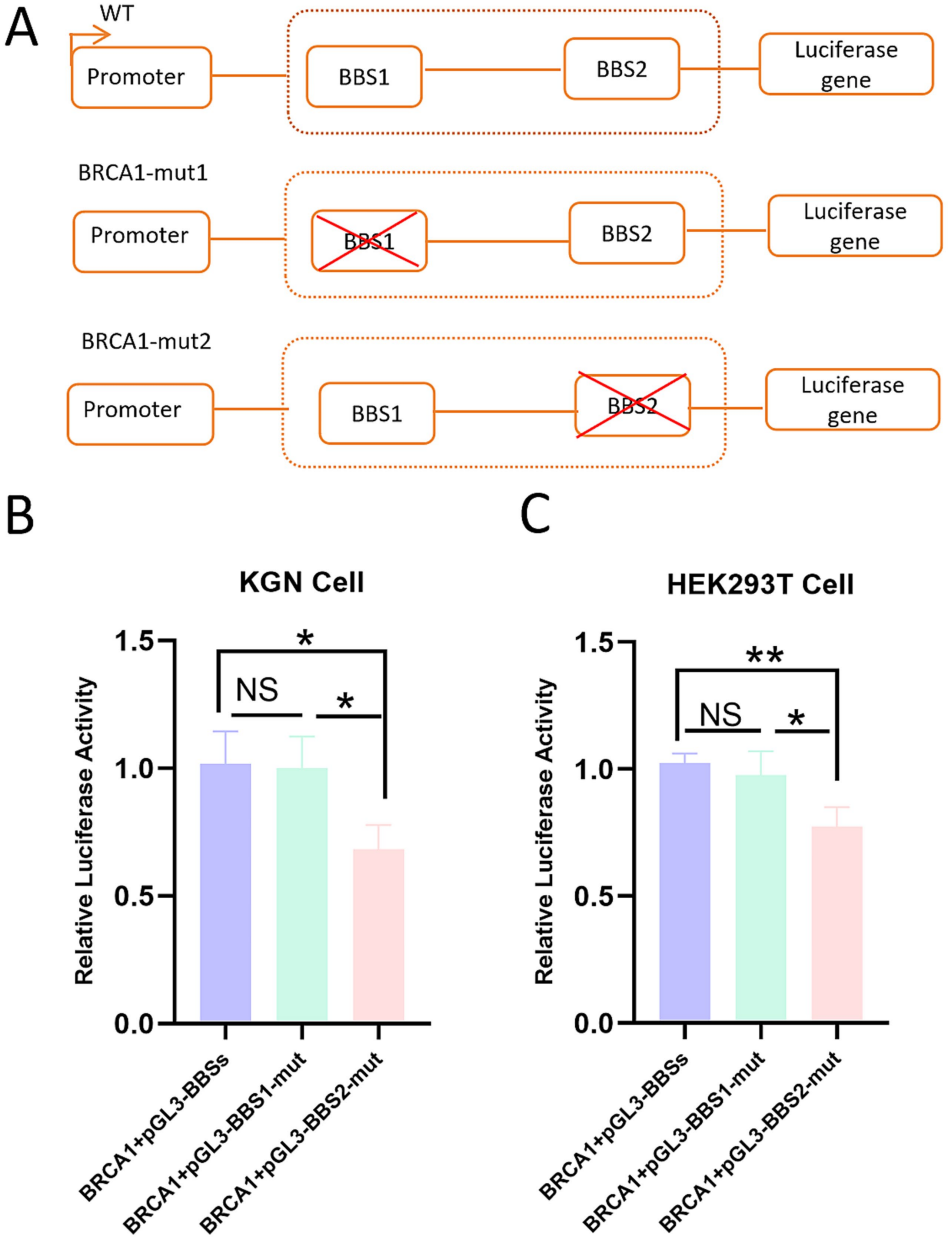
BRCA1 exerts its regulatory effect on the transcription of the BMPR1B by acting through the BSS2 locus. **(A)** Reporter constructs of wild-type and mutant BRCA1-binding sites. Effect of BRCA1 on the promoter activity of the BSS1-mutant reporter vector in KGN and HEK293T cells **(B,C)**. Effect of BRCA1 on the promoter activity of the BSS2-mutant reporter vector in KGN cells **(B)** and HEK293T cells **(C)**. Each bar represents the mean ± SEM (*n* ≥ 3). *p* ≤ 0.01, *p ≤ 0.05, NS indicates not significant.

To elucidate which specific BBSs modulate the transcriptional activity of the *Ovis aries* BMPR1B promoter, we generated a reporter vector construct harboring a single BBS mutation within the BMPR1B promoter. We performed site-directed mutagenesis on the two BRCA1-binding sites (BBS1 and BBS2), respectively, as shown in Figure 4A. Transcriptional activity was assessed through a reporter assay, performed by co-transfecting KGN and HEK293T cells with both the reporter plasmid and the pcDNA3.1-BRCA1 overexpression vector, and subsequently measuring luciferase activity. The results demonstrated that mutation of the BBS1 site did not significantly affect BMPR1B promoter activity upon BRCA1 overexpression in both KGN and HEK293T cells. In contrast, mutation of the BBS2 site markedly reduced promoter activity, highlighting its critical role in this process(Figures 4B,C). These findings precisely identify BBS2 as the primary functional site for BRCA1-dependent BMPR1B transactivation.

### 3.5 The expression of the BMPR1B gene is up-regulated by the transcription factor BRCA1 in ovine ovarian GCs

**Figure 5:**
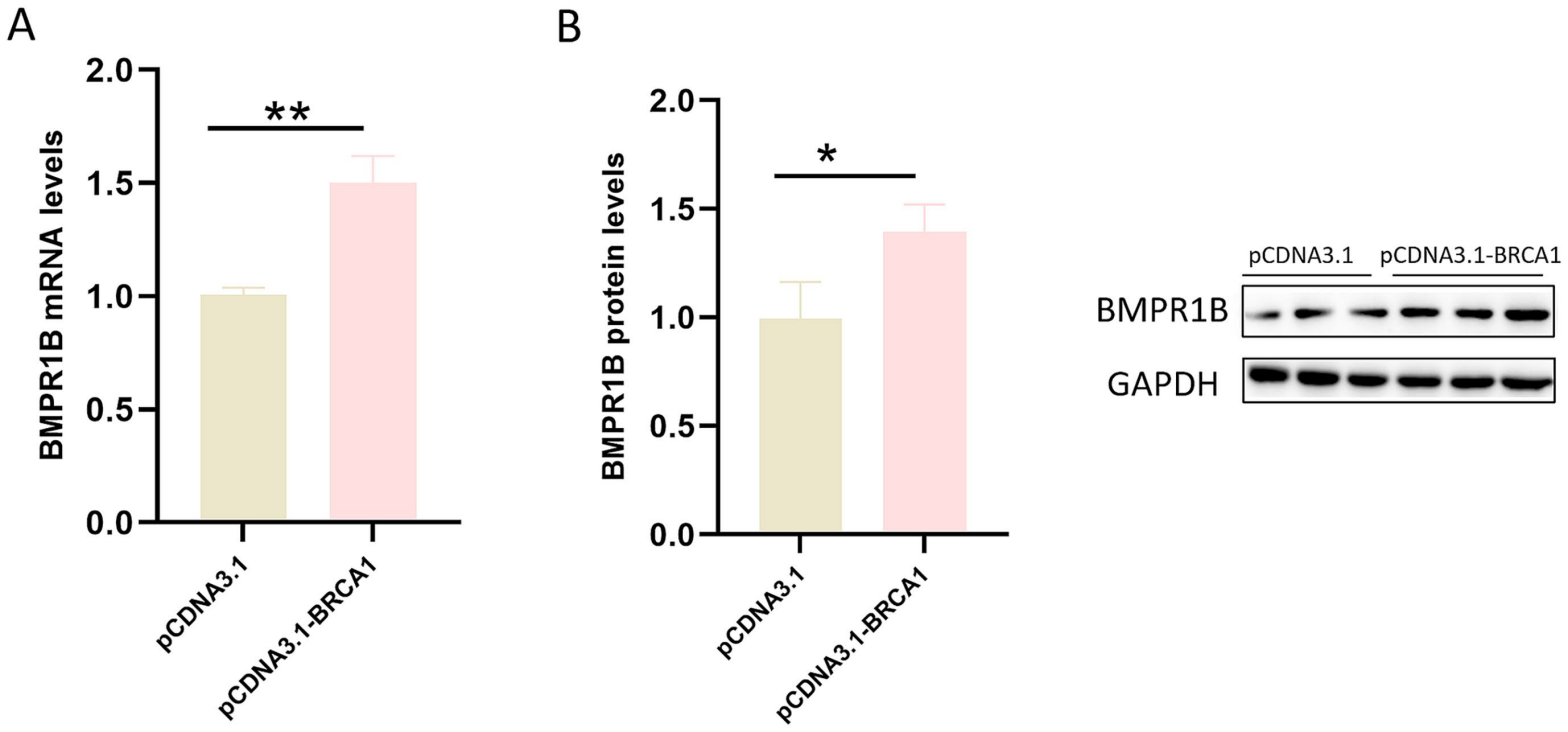
BMPR1B expression is up-regulated in ovine GCs following BRCA1 overexpression. **(A)** Shows the relative mRNA expression levels, while **(B)** displays the corresponding protein expression levels; each bar represents the average ± SEM (*n* ≥ 3). ^**^p ≤ 0.01, ^*^p ≤ 0.05.

The objective of this study was to assess how BRCA1 regulates BMPR1B expression in ovine ovarian GCs. To this end, we transfected primary GCs cultured *in vitro* with the BRCA1 overexpression plasmid, pcDNA3.1-BRCA1. Subsequently, BMPR1B mRNA levels were quantified by quantitative real-time PCR (qRT-PCR) and normalized to an internal reference gene. The relative expression in BRCA1-overexpressing cells was compared with that of cells transfected with the empty pcDNA3.1 vector. As shown in Figure 5A, BMPR1B mRNA expression was markedly higher in GCs following BRCA1 overexpression than in control cells. This result indicates a role for BRCA1 as a transcriptional activator of BMPR1B in ovine ovarian GCs. Western blot analysis further revealed that, compared with the control, the BRCA1-overexpressing group exhibited a marked increase in BMPR1B protein levels within ovarian GCs (Figure 5B).

### 3.6 The apoptosis of sheep ovarian GCs is inhibited by the transcription factor BRCA1

**Figure 6:**
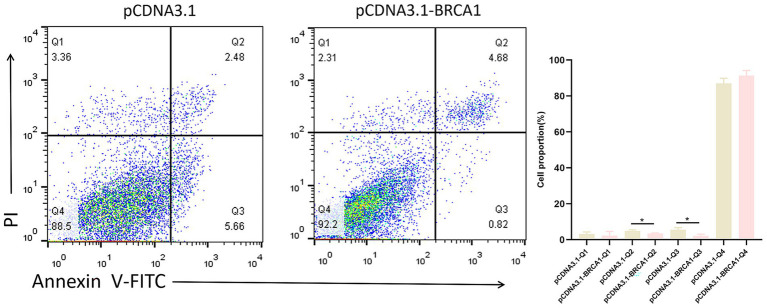
BRCA1 effectively prevents apoptosis in ovine GCs. Ovine GCs were transfected with the BRCA1 expression vector pcDNA3.1-BRCA1, and their apoptosis rate was subsequently measured using FACS. Each bar represents the average ± SEM (*n* ≧ 3); *p ≤ 0.05.

The apoptotic rate of *in vitro*-cultured primary ovine ovarian GCs was assessed by flow cytometry (FACS) following transfection with a BRCA1 overexpression vector (pcDNA3.1-BRCA1) to analyze the gene’s role in apoptosis. Transfection with the BRCA1 overexpression vector significantly decreased the apoptosis rate in GCs relative to the control group. According to this discovery, apoptosis is suppressed by the BRCA1 gene in ovarian GCs of sheep; the results are presented in Figure 6.

## 4 Discussion

While the role of BMPR1B in ovarian function is well-established, the transcriptional mechanisms governing its expression remain poorly understood. Here, we uncover a direct transcriptional pathway in which the transcription factor BRCA1 binds to the BBS2 site within the BMPR1B promoter, activating its expression and thereby inhibiting granulosa cell (GC) apoptosis. This reveals a novel, non-canonical role for BRCA1 in ovarian physiology, distinct from its DNA repair function.

Our discovery was initiated by bioinformatic analysis using online prediction tools that identified putative binding sites for several transcription factors, including BRCA1, KLF4, and SP1, within this promoter region. Through luciferase assays, promoter activity was found in the P1 fragment, a region where two BRCA1 binding sites (BBS1 and BBS2) had been predicted. Interestingly, while two binding sites were predicted, only BBS2 was confirmed as a functional site, suggesting that the presence of chromatin background or co-transcription factors may determine the binding specificity of BRCA1. Based on our findings and the well-documented high expression and essential DNA repair role of BRCA1 in ovarian tissue (32), we hypothesize that BRCA1 performs two protective functions in ovarian granulosa cells: one involves upholding genomic stability via DNA repair, and the other, as our data implies, involves stimulating the transcription of survival genes such as BMPR1B. Consequently, if the BRCA1 function is impaired, it might cause a double failure by weakening DNA repair and compromising BMP-mediated survival signaling, thereby collectively reducing the apoptotic threshold. The exact molecular process by which BRCA1’s attachment to BBS2 controls gene transcription is still being investigated. Our experimental data support a verifiable theory: the BRCT domain within BRCA1 might function as an anchoring point for chromatin-modifying complexes once it attaches to DNA. Such a model could mechanistically explain how BRCA1 affects its chromatin surroundings to initiate transcription. Additionally, the predicted simultaneous binding of KLF4 and SP1 within the P1 fragment implies potential cooperative regulation, necessitating further research.

The BMPR1B gene is presently recognized as the principal fecundity gene that influences ovulation and litter size. Its classic FecB mutation (c.746A > G,p. Q249R) enhances the sensitivity of GCs to BMP signaling, thereby accelerating follicular maturation and resulting in an increase of 1–2 lambs per ovulation event. Ewes heterozygous for the mutation exhibit an average increase of 0.7 lambs per litter, whereas homozygous ewes show an average increase of 1.2 lambs per litter. Ewes carrying one copy of this gene have an average birth rate of 0.7 additional lambs, while those with two copies can produce 1.2 more (33). The BMPR1B mutation is highly prevalent in prolific ovine breeds such as the Small-Tailed Han, Hu, and Dorset, thereby serving as a principal molecular marker for breeding programs aimed at enhancing multiple-birth traits in sheep worldwide. This study reveals that BRCA1 transcriptionally activates BMPR1B, representing a regulatory layer that operates in parallel with the FecB mutation. FecB contributes to higher ovulation numbers through heightened receptor sensitivity. Simultaneously, BRCA1, by operating as a transcription factor, elevates the expression of the BMPR1B gene, thereby indirectly intensifying BMP signaling to foster follicle survival and development. The two work together to determine the final output of a functional follicle pool. Furthermore, posttranscriptional regulation of BMPR1B (such as miR-1306 targeting its 3′-UTR to promote apoptosis) and transcriptional regulation (such as Smad4 co-activation) together constitute a finely tuned dose regulation network (6, 7). BRCA1 transcriptional activation acts as a “brake” in this network, preventing BMPR1B levels from falling below the survival threshold. This multi-layered regulatory model explains why small changes in BMPR1B expression can significantly affect reproductive phenotype.

This study provides novel insights into the transcriptional regulation of BMPR1B by BRCA1 in ovine ovarian GCs, yet several limitations warrant acknowledgment. First, the main limitation lies in verifying the identity of granulosa cells. Although the cells were isolated according to established protocols and selected based on standard morphological criteria consistent with primary granulosa cells in culture, this approach is still not ideal. Morphological assessment alone cannot definitively distinguish granulosa cells from other ovarian epithelial cell types. The most rigorous verification requires immunofluorescence staining for granulosa cell-specific markers (such as FSHR and CYP19A1). Unfortunately, due to current technological limitations, we were unable to perform this decisive characterization. Future research will prioritize immunofluorescence or other specific immunochemical methods to definitively confirm the purity and identity of the cell population, thereby solidifying the foundation of the experimental model. Second, although luciferase reporter assays and site-directed mutagenesis strongly suggest that BRCA1 modulates BMPR1B transcription through direct binding to the BBS2 site within the promoter, direct physical evidence of this interaction is lacking. Definitive confirmation could be achieved by employing chromatin immunoprecipitation (ChIP) or electrophoretic mobility shift assays (EMSA). Third, the initial characterization of BMPR1B promoter activity was performed using human-derived cell lines (HEK293T and KGN) rather than primary ovine GCs. While these cell lines offer practical advantages such as high transfection efficiency and experimental reproducibility, they may not fully recapitulate the species-specific transcriptional milieu. Based on the limitations identified, future studies will focus on three key directions: implementing immunofluorescence with ovine-specific markers to definitively validate granulosa cell identity; employing ChIP assays to provide direct evidence of BRCA1 binding to the BMPR1B promoter; and establishing optimized transfection protocols for conducting promoter analyses directly in primary ovine granulosa cells. Addressing these methodological challenges will strengthen the experimental foundation and enhance the physiological relevance of future findings in this research area.

## 5 Conclusion

Overall, this study implies that the Hu sheep BMPR1B gene’s promoter region is rich in prospective regulatory elements. Moreover, our experimental findings reveal that the transcription factor BRCA1 is linked to increased BMPR1B expression in ovarian GCs and leads to a significant reduction in programmed cell death in granulosa cells. Taken together, these results provide valuable understanding of the possible molecular mechanisms that determine granulosa cell destiny and lay the groundwork for future investigations into ovarian function.

## Data Availability

The original data supporting the conclusions of this article will be provided by the author without reservation.
