# Supplementary material for: Transcription factor BRCA1 is associated with BMPR1B transcriptional activity in sheep ovarian granulosa cells
Source: Front Vet Sci. 2026 Jan 14;12:1722859. doi: 10.3389/fvets.2025.1722859 (PMC12853376; doi:10.3389/fvets.2025.1722859)
Supplement: Supplementary file 1 [file Table_1.DOCX]

**Table 1.** The primer sequences

| **Primer** | **Gene** | **Primer Sequence (5'-3')** | **Usage** | **Size(bp)** |
| --- | --- | --- | --- | --- |

| Primer1 |  | BMPR1B | | | F: GAGCTCAGCTGGGTCAGGCTCCTTTC | | Deletion construction | | 416 |
| --- | --- | --- | --- | --- | --- | --- | --- | --- | --- |
|  |  |  | | | R: CTCGAGAGACTCCTCTGCTGCCACTC | |  | |  |
| Primer2 |  | BMPR1B | | | F: GAGCTCTTCCCAGCATGAATCAGAGTC | | Deletion construction | | 975 |
|  |  |  | | | R: CTCGAGAGACTCCTCTGCTGCCACTC | |  | |  |
| Primer3 |  | BMPR1B | | | F: GAGCTCGGAACTGAGGATGTTGGATTG | | Deletion construction | | 1485 |
|  |  |  | | | R: CTCGAGAGACTCCTCTGCTGCCACTC | |  | |  |
| Primer4 |  | BMPR1B | | | F: GTATAGGTGTGTGTCTCTCCGGAATG  CCTCCACAGCTGCCCA | | Mutation construction | | 308 |
|  |  |  | | | R: GACCACAGGCATCCCAGA | |  | |  |
| Primer5 |  | BMPR1B | | | F: AGCACTCAAGGCAAACCA | | qPCR | | 509 |
|  |  |  | | | R: GGCCATGATGTAAGACTGAAAG | |  | |  |
| Primer6 |  | | GAPDH | | | F: TGGAATGACATCTCGGTCTGGTA | qPCR | | 525 |
|  |  | | |  | | R: CACCATGGCTCAGAAGCACAC | |  |  |
